# Identification of QTL for branch traits in soybean (*Glycine max* L.) and its application in genomic selection

**DOI:** 10.3389/fgene.2025.1484146

**Published:** 2025-03-03

**Authors:** Qichao Yang, Jing Wang, Yajun Xiong, Alu Mao, Zhiqing Zhang, Yijie Chen, Shirui Teng, Zhiyu Liu, Jun Wang, Jian Song, Lijuan Qiu

**Affiliations:** ^1^ MARA Key Laboratory of Sustainable Crop Production in the Middle Reaches of the Yangtze River (Co-construction by Ministry and Province), College of Agriculture, Yangtze University, Jingzhou, China; ^2^ The Shennong Laboratory, Zhengzhou, Henan, China; ^3^ State Key Laboratory of Crop Gene Resources and Breeding, Institute of Crop Sciences, Chinese Academy of Agricultural Sciences, Beijing, China; ^4^ National Key Facility for Gene Resources and Genetic Improvement / Key Laboratory of Crop Germplasm Utilization, Ministry of Agriculture and Rural Affairs / Institute of Crop Sciences, Chinese Academy of Agricultural Sciences, Beijing, China; ^5^ National Nanfan Research Institute (Sanya), Chinese Academy of Agricultural Sciences, Sanya, Hainan, China

**Keywords:** soybean, branch, QTL, candidate gene, genomic selection

## Abstract

**Introduction:**

Branches are important for soybean yield, and previous studies examining branch traits have primarily focused on branch number (BN), while research assessing branch internode number (BIN), branch length (BL), and branch internode length (BIL) remains insufficient.

**Methods:**

A recombinant inbred line (RIL) population consisting of 364 lines was constructed by crossing ZD41 and ZYD02878. Based on the RIL population, we genetically analyzed four branch traits using four different GWAS methods including efficient mixed-model association expedited, restricted two-stage multi-locus genome-wide association analysis, trait analysis by association, evolution and linkage, and three-variance-component multi-locus random-SNP-effect mixed linear model analyses. Additionally, we screened candidate genes for the major QTL and constructed a genomic selection (GS) model to assess the prediction accuracy of the four branch traits.

**Results and Discussion:**

In this study, four branch traits (BN, BIN, BL, and BIL) were phenotypically analyzed using the F_6_-F_9_ generations of a RIL population consisting of 364 lines. Among these four traits, BL exhibited the strongest correlation with BIN (0.92), and BIN exhibited the strongest broad-sense heritability (0.89). Furthermore, 99, 43, 50, and 59 QTL were associated with BN, BIN, BL, and BIL, respectively, based on four different methods, and a major QTL region (Chr10:45,050,047..46,781,943) was strongly and simultaneously associated with all four branch traits. For the 207 genes within this region, nine genes were retained as candidates after SNP variation analysis, fixation index (*F*_*ST*_), spatial and temporal expression analyses and functionality assessment that involved the regulation of phytohormones, transcription factors, cell wall and cell wall cellulose synthesis. Genomic selection (GS) prediction accuracies for BN, BIN, BL, and BIL in the different environments were 0.59, 0.49, 0.48, and 0.56, respectively, according to GBLUP. This study lays the genetic foundation for BN, BIN, BL, and BIL and provides a reference for functional validation of regulatory genes in the future.

## 1 Introduction

Soybeans (*Glycine max* L. Merrill) are rich in protein and oil and are one of the major global crops in the world, and they play a key role in the food and feed industries. The soybean branch is an important component of the soybean plant and affects soybean yield (Guo and Guo, 2021; Li et al., 2021). Furthermore, branch yield was much higher than was main stem yield in certain soybean cultivars (Board, 1987). Soybean branch traits affect soybean yield by influencing photosynthesis and assimilation product distribution in the aboveground canopy of soybeans and are positively correlated with soybean yield per plant (Li et al., 2011; Qiao et al., 2016). The effective branch number (BN), branch internode number (BIN), branch length (BL), and branch internode length (BIL) were the four subtraits of soybean branches (Guo et al., 2015; Qiao et al., 2016; Carpenter and Board, 1997). Modern cultivars have been domesticated from wild soybeans (*Glycine soja* Sieb. and Zucc.); however, many traits were attenuated during this process. For example, high protein content and stress resistance due to a reduction in genetic diversity and the utilization of elite traits from wild soybeans have both become valid approaches to improve modern cultivars (Jin et al., 2003; He et al., 2014; Nawaz et al., 2018; Wang et al., 2019).

Genetically, BN, BIN, BL, and BIL are controlled by multiple loci, and 21 quantitative trait locis (QTL) associated with branching have been identified on chromosomes 4, 5, 6, 10, 11, 12, 14, 15, 17, 18, and 19 (https://www.soybase.org/). The branching-related QTL *qBN-1* on chromosome 6 (R^2^ = 22.69%) was identified in the segregating populations of high-branching and low-branching cultivars (Lamlom et al., 2020). Using cultivars from different genetic backgrounds and 7,189 single nucleotide polymorphisms (SNPs), the branch number-related QTL *qBR6_1* was mapped to chromosome 6 near the *E1* gene, which controls flowering time, thus suggesting a possible pleiotropic effect for *E1* (Yang et al., 2017). Among the ten QTL associated with branch number that were identified in the three populations, QTL-*qBN.C2* (R^2^ = 33.3%) was identified as the major QTL. *Glyma.06G188400* within this QTL may participate in branching development by regulating the axillary meristematic tissue (Yang et al., 2021). The gene *Glyma.06G210600* located on *qBR6-1* was believed to be a candidate gene for branch number, as it encodes a TEOSINTE-BRANCHED1/CYCLOIDEA/PROLIFERATING CELL FACTORS 1 and 2 (TCP) transcription factor that may be involved in the regulatory network of branching and growth (Shim et al., 2017; Shim et al., 2019). Overexpression of *GmmiR156b* increased the number of branches and resulted in decreased expression of *GmSPL9*, thus indicating that the GmmiR156-SPL module is involved in branching regulation (Bao et al., 2019; Sun et al., 2019). The ectopic expression of *GmMYB181*, a soybean R2R3-MYB transcription factor, in *Arabidopsis* increases branch number, possibly through shoot development or hormone signaling pathways (Yang et al., 2018). Additionally, *SoyZH13_18g242900*, also known as *Dt2*, was highly expressed in the lateral shoots and shoot tips (Liang et al., 2022). *Abr11* was localized as a QTL associated with the average BL (La, 2018). Nonetheless, seldom studies have been focused on BIN and BIL.

Genomic selection (GS) is an effective tool for improving breeding selection efficiency and shortening the breeding cycle, and it has been widely applied in the context of quantitative traits of both animals and crops (Duhnen et al., 2017). Genomic prediction accuracy is affected by additive effects, model selection, population type, marker density, gene effects, heritability, genetic architecture, and the extent and distribution of linkage disequilibrium (LD) between markers and QTL (Desta and Ortiz, 2014). Among these GS methods, the random regression best linear unbiased predictor (rrBLUP) and genomic best linear unbiased predictor (GBLUP) are important and popular (Rabier et al., 2016). In soybeans, the phenotypic variations in seed protein content and yield were predicted using the GBLUP model, and it was observed that the modeling of additive-by-additive epistasis possessed a higher prediction accuracy than did the modeling of additive effects (Duhnen et al., 2017). Additionally, the genomic selction of soybean proteins using different SNPs revealed that the prediction accuracy was higher when using major SNPs or when increasing the density of SNPs (Qin et al., 2022).

In this study, a recombinant inbred line (RIL) population derived from a cross between Zhongdou 41 (ZD41) and ZYD02878 was used to explore the genetic mechanisms of four branch traits, e.g., branch number (BN), branch internode number (BIN), branch length (BL), branch internode length (BIL), in soybeans. A total of 251 different QTL related to those four branch traits were identified using four different methods, and possible candidate genes were analyzed. Based on these results, the prediction accuracies of these traits were estimated and compared. Our results provide a solid foundation for elucidating these genetic mechanisms.

## 2 Materials and methods

### 2.1 Plant materials

In the summer of 2015, ZD41 (♀) and ZYD02878 (♂) were crossed and yielded a hybrid population at the Crop Science Experimental Base of Yangtze University in Jingzhou City, Hubei Province (112.06°E, 30.37°N), and a RIL population consisting of 364 lines was constructed by single seed descent (SSD) from the F_2_-F_5_ generations (Chen et al., 2023).

The F_6_-F_8_ RIL population was planted during 2019–2020 in Jingzhou (JZ) and Sanya (SY) according to a previous report (Chen et al., 2023), and F_9_ was planted in 2021 at Ajian Farm, Zhengji Township, Shangqiu, Henan Province (115.98°E,34.41°N) from June to October. Hereafter, the four different environments are designated 19JZ, 19SY, 20JZ, and 21SQ.

### 2.2 Phenotyping and statistics

The effective BN of each plant was scored according to the Descriptors and Data Standards for Soybeans (*Glycine* spp.) (Qiu and Chang, 2006). The BIN and BL values of each effective branch were measured. BIL data are obtained from the raw data of the BL and BIN that are presented as BL/BIN. BN was scored in four environments (19JZ, 19SY, 20JZ, and 21SQ), and three environments (19JZ, 19SY, and 20JZ) were used for BL, BIN, and BIL scoring. The data for each environment consisted of two parents and a RIL populations consisting of 364 lines.

The 1.5×interquartile range (IQR) and 3-σ principle were applied to exclude the outliers when the mean values for BL, BIN, and BIL of an individual plant, technical replicates of each RIL line, and biological replicates of different environments were calculated.

Descriptive statistics, correlation, and normality analyses were performed using the R package lme4, and the broad-sense heritability (*h* ^*2*^) was calculated as described by Chen et al. (2023).

### 2.3 Genotyping and genetic analysis

Genomic DNA was isolated from young fresh leaves using cetyltrimethylammonium bromide (CTAB) method (Rogers and Bendich, 1988), and then “ZDX1” (an Illumina soybean 200 K gene chip) was adopted for genotyping (Sun et al., 2022). Raw genotype was filtered according to MAF <0.05 and integrity >0.8 using PLINK2.0 (Wen et al., 2014), and resulted in a total of 117,772 high quality SNPs. Then, 6,098 bin markers were constructed using SNPbinner and high-quality SNPs (Gonda et al., 2019).

To identify QTL associated with BN, BIN, BL, and BIL, four different methods were used. These included Efficient Mixed-Model Association expedited (EMMAX) (Xue et al., 2013), Restricted Two-stage Multi-locus Genome-Wide association analysis (RTM-GWAS) (He and Gai, 2020), Trait Analysis by association, Evolution and Linkage (TASSEL), and three-variance-component multi-locus random-SNP-effect mixed linear model analyses (3VmrMLM) (Leamy et al., 2017; Zuo et al., 2022).

The kinship matrix (relatedness) was calculated using emmax-intel64 based on 6,098 bin markers and was applied to correct for population structure and relatedness in the mixed linear models (Zhao et al., 2020). For GWAS analysis using TASSEL, the first 20 principal components (PCs) was calculated using GCTA, kinship was calculated using TASSEL, and both the 20 PCs and kinship were introduced as covariates in the association study using the Mixed Linear Model (MLM). Based on the Bonferroni method, the significance threshold for EMMAX and RTM-GWAS was determined to be 1/m, where m is the number of SNPs. To perform RTM-GWAS, 4,715 SNP linkage disequilibrium blocks (SNPLDBs) were first inferred with a MAF of 0.01 and a maximum block length of 10,000. The GSC matrix was then calculated for QTL detection. In order to balance false positives and false negatives in hypothesis testing, RTM-GWAS commonly use 0.01 as the statistical significance level to control the whole test error rate. Based on suggestion of 3VmrMLM method developer, single-environment analysis was performed using a 3 variance-component multi-locus random SNP effects mixed linear model constructed using 3VmrMLM with CriLOD = 3 to identify significantly associated bins. Besides, QTL regions of EMMAX and TASSEL are defined by 100 kb up and downstream of the significantly associated SNPs.

Based on the identified QTL, the LD between QTL was calculated using PLINK and R, and QTL with an average >0.9 in the region were recognized as co-localized QTL. Unique QTL are contiguous regions of co-localized QTL that are strung together by multiple adjacent or overlapping co-localized QTL.

### 2.4 Candidate gene identification

The QTL regions most likely associated with branch traits were identified by analyzing the frequency and effects of the mapped QTL. The significance of EMMAX was subsequently set to 0.01 to narrow the QTL region. Genes within associated regions were first annotated by SoyBase (https://soybase.org) and phytozome (https://phytozome-next.jgi.doe.gov/info/Gmax_Wm82_a2_v1). Single nucleotide polymorphism (SNP) variation, genetic differentiation analysis, and temporal and spatial tissue-specific gene expression pattern analysis were performed to narrow the candidate gene list. For genetic differentiation analysis, the fixation index (*F*_*ST*_) was calculated from published genome sequence data using vcftools (0.1.13) with a 100 bp window size, and potentially domesticated genes were defined if the *F*_*ST*_ within coding regions was larger than 0.6 (Danecek et al., 2011; Song et al., 2013; Li et al., 2023). Gene expression data regarding axillary meristem, flower, leaf, meristem, nodule, pod, root, root hair, shoot apex and shoot apical meristem (SAM) were extracted from the PPRD database, and heatmaps were constructed using the R package ‘pheatmap’ (Shim et al., 2017; Shim et al., 2019).

### 2.5 Genomic prediction

Based on the bin markers and associated markers using four different methods, three different marker sets were constructed. These included (1) 6,098 genome-wide markers (GWM), (2) 400 trait-associated markers (TAM), and (3) 5,698 genome-wide unassociated markers (GWUM) (Chen et al., 2023). The trait-associated markers are SNP markers mapped genome-wide by the QTL for the branch-associated traits that we have located, and because of the overlap and size of the regions between the QTL that we have located, these QTL cover a total of 400 SNP regions. To compare the effects of trait-associated regions on GS precision, three marker sets in the GBLUP model were used as random effects, and a five-fold cross-validation and 20 replications were performed to measure the average prediction precision of each GS model. To ensure the reproducibility of the results, the same random seeds were used in 20 replicates. The GBLUP model was constructed using the R package rrBLUP.

## 3 Results

### 3.1 Phenotype analysis of BN, BIN, BL and BIL

**FIGURE 1:**
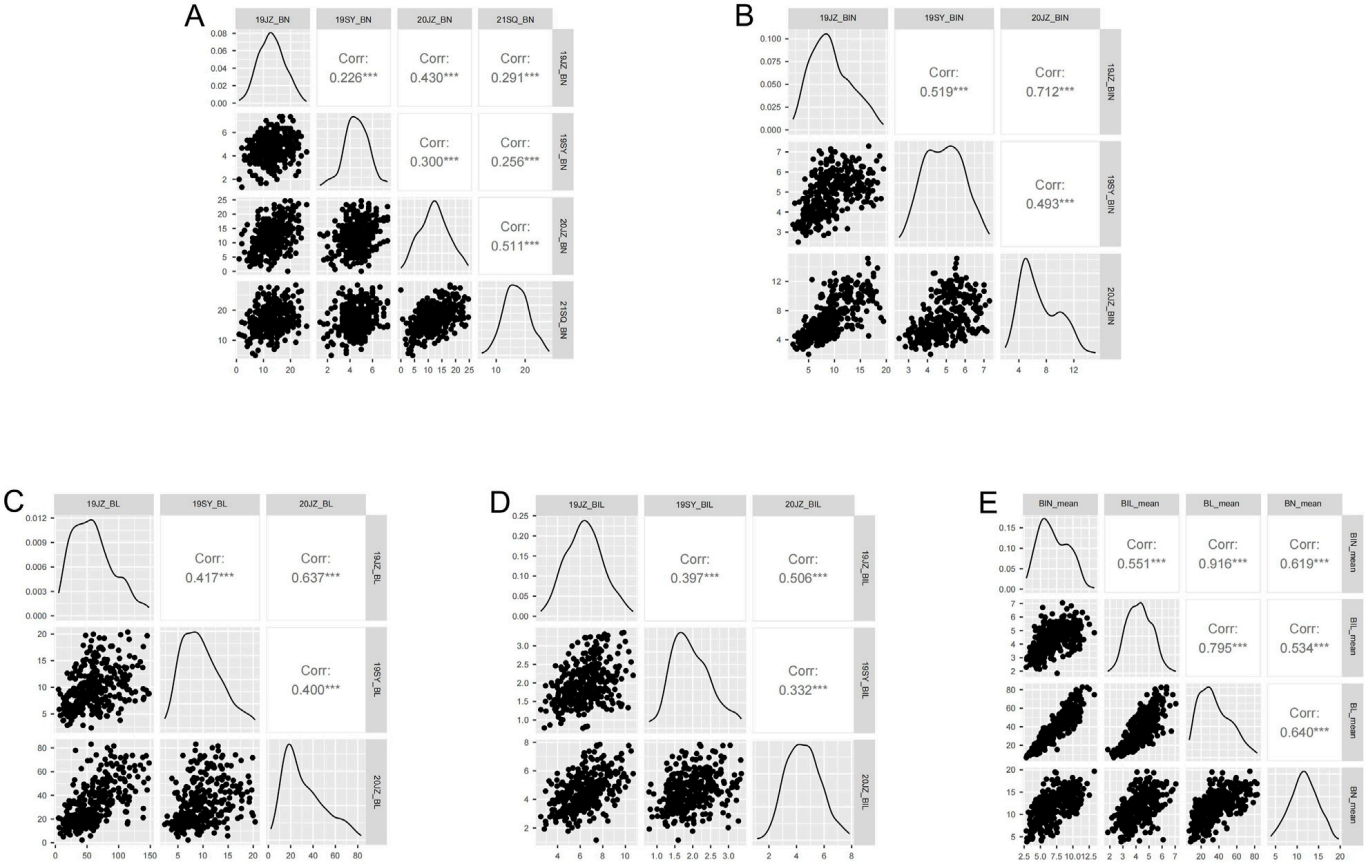
Correlation analysis of BN, BIN, BL, and BIL in different environments. **(A–E)** correlation of BN, BIN, BL, BIL, and mean value of different environments. *, **, *** represents significance level of 0.05, 0.01, and 0.001 respectively.

Based on the 3-σ principle and 1.5×interquartile range (IQR), the outliers of BN, BIN, BL, and BIL and the mean in each environment were removed and analyzed with descriptive statistics, and it was observed that normal or nearly normal distribution was observed in the RIL population regarding the four traits in different environments (Table 1; Figure 1; Supplementary Figure S1). For each trait, BN, BIN, BL, and BIL values ranged between 0-28.33, 2.00-19.78, 2.30–152.66 cm, and 0.78–10.69 cm, respectively. Transgressive segregation occurs universally for different traits under different environments. In general, considerably more individuals exceeded the higher-value parent than they did the lower-value parent in BN, BIN, and BL; however, an opposite trend was observed for 19JZ and 19SY of BIL and 21SQ of BN. Correlation analysis revealed significant correlations among all four traits (BN, BIN, BL, and BIL). BIN and BL exhibited the highest correlation (Pearson’s coefficient r = 0.92, P < 0.001), and this was followed by BL and BIL (r = 0.80, P < 0.001) and BN and BL (r = 0.64, P < 0.001). The lowest correlation was observed between BN and BIL (Figure 1E). Additionally, all four traits were significantly correlated in each environment; however, the four traits in 19SY were less correlated with other environments (Figures 1A–D). The BIN in different environments exhibited the highest correlation with BN, BL, and BIL (Figures 1A–D). The broad-sense heritability of BN, BIN, BL, and BIL was 0.88, 0.89, 0.75, and 0.85 (Table 2), respectively, thus indicating that the environment exerted greater impact on BL than it did on the other traits.

**TABLE 1:** Descriptive statistics of BN, BIN, BL, and BIL in different environments.

Trait	Environment	Parents			RIL							TSELP	TSEHP	NA	Outlier
		P1	P2	|P1-P2|	Max	Min	Range	Mean	SD	Skewness	Kurtosis				
BN	19JZ	3.00	7.00	4.00	26.00	1.00	25.00	13.00	4.74	0.18	−0.30	27	290	7	4
	19SY	3.67	1.33	2.33	7.33	1.33	6.00	4.54	1.10	−0.09	0.07	0	268	3	4
	20JZ	3.67	13.50	9.83	24.67	0.00	24.67	12.08	5.36	0.11	−0.58	18	138	1	3
	21SQ	6.67	34.33	27.67	28.33	5.00	23.33	17.09	4.42	0.13	−0.28	2	0	13	3
	mean	5.50	14.04	8.54	19.67	3.89	15.78	11.63	3.16	−0.04	−0.35	10	82	0	3
BIN	19JZ	4.50	7.29	2.79	19.78	2.00	17.78	9.38	3.89	0.50	−0.47	33	237	3	1
	19SY	2.83	3.25	0.42	7.29	2.50	4.79	4.89	1.02	0.08	−0.79	1	340	4	2
	20JZ	3.06	6.35	3.29	15.16	2.00	13.16	7.01	2.74	0.60	−0.63	6	180	2	1
	mean	3.46	5.63	2.16	13.30	2.84	10.46	7.11	2.28	0.35	−0.75	7	247	0	0
BL (cm)	19JZ	23.39	70.26	46.87	152.66	5.10	147.56	62.37	34.09	0.57	−0.42	47	127	3	6
	19SY	9.26	4.40	4.86	20.49	2.36	18.12	9.88	3.93	0.56	−0.30	9	175	4	12
	20JZ	6.67	41.94	35.27	83.19	2.30	80.89	32.79	18.67	0.79	−0.32	4	103	2	15
	mean	13.10	38.86	25.76	83.18	6.77	76.41	35.81	17.67	0.57	−0.46	24	136	0	5
BIL (cm)	19JZ	5.28	9.61	4.33	10.69	2.24	8.45	6.43	1.66	0.13	−0.35	93	13	3	4
	19SY	2.99	1.37	1.63	3.36	0.78	2.58	1.95	0.54	0.46	−0.15	41	17	4	8
	20JZ	2.02	6.16	4.14	7.85	1.14	6.71	4.52	1.28	0.22	−0.21	5	39	2	3
	mean	3.43	5.71	2.28	7.05	1.84	5.21	4.31	0.96	0.19	−0.33	73	22	0	4

P1: ZD41; P2: ZYD02878; BN: branch number; BIN: branch internode number; BL: branch length; Max: maximum; Min: minimum; SD: standard deviation; JZ: jingzhou; SY: sanya; SQ: shangqiu; TSEHP: transgressive segregation exceeds higher parent; TSELP: transgressive segregation exceeds lower parent.

**TABLE 2:** Variance components and broad sense heritability of different traits.

Trait	h^2^	$\sigma_{g}^{2}$	$\sigma_{gly}^{2}$
BN	0.88	5.67	3.35
BIN	0.89	2.10	1.99
BL	0.75	89.71	241.20
BIL	0.85	0.50	0.66

$h^{2}$ : broad sense heritability; $\sigma_{g}^{2}$ : genotype variance; $\sigma_{\text{gly}}^{2}$ : a three-level interaction variance of genotype, year, and location.

### 3.2 Genetic analysis of branch related traits

A total of 12, 3, 3, and 4 unique QTL regions that were significantly associated with BN, BIN, BL, and BIL, respectively, were identified by EMMAX using the phenotypes of the different environments (19JZ, 19SY, 20JZ, and 21SQ, respectively) and the means (Supplementary Table S4). These QTL were distributed on eight different chromosomes (2, 3, 7, 10, 11, 12, 13, and 19) and were predominantly concentrated on chromosomes 10 and 19, and they accounted for 64.10% of the total significant regions (Supplementary Table S1). Of all QTL identified in the different environments, the peak SNP of the most significant QTL region Chr12:2,621,904..5,817,926 for BN was Chr12: 5,396,475 with a -log_10_(P) of 6.59 (Supplementary Figure S2; Supplementary Table S2; Supplementary Table S4). Another QTL on chromosome 10 (Chr10:43,366,437..46,985,577) was determined to be not only significantly associated with BIN but also with BL, and both shared the same peak SNP of Chr10:45, 290, 023 (Supplementary Figure S2; Supplementary Table S2; Supplementary Table S4). This region for BIN and BL was repeatedly identified at 19 JZ, 20 JZ, and at the mean (Supplementary Table S1). Among the QTL associated with BIL, the major QTL was mapped to the region of Chr13:37,175,077..39,002,308, within which the peak SNP was Chr13:38, 260, 409 with a -log_10_(P) of 5.65 (Supplementary Figure S2; Supplementary Table S2; Supplementary Table S4).

A total of 11 significant QTL regions (six, two, two, and one QTL for BN, BIN, BL, and BIL, respectively) as detected by TASSEL were distributed on six chromosomes (3, 10, 11, 12, 13, and 19) (Supplementary Table S4). Similar to the QTL identified by EMMAX, most of the QTL identified by TASSEL were located on chromosomes 10 and 19. Among the six QTL associated with BN, the most significant was Chr12:3,633,162..6,216,535 that was identified from the mean and yielded a phenotypic variance explained (PVE) of 7.18% (Supplementary Figure S3; Supplementary Table S1; Supplementary Table S4). The QTL regions for BL were located on chromosomes 10 and 11, and the region on chromosome 10 (Chr10:42,222,333..45,658,686) was identified by 20JZ (Supplementary Table S1; Supplementary Table S4). The mean was also mapped by BIN (Supplementary Table S1; Supplementary Table S4). For BIL, only one region of Chr13:37,360,709..39,002,308 was identified by TASSEL from 20 JZ and the mean; however, this region was not identified in BN, BIN, or BL (Supplementary Table S1; Supplementary Table S4).

Based on 4,715 SNPLDBs, 51, 22, 30, and 39 unique QTL associated with BN, BIN, BL, and BIL, respectively, were identified on all 20 chromosomes (Supplementary Table S4). The PVE of each QTL ranged from 1.54% to 21.06%, and this explained 26.02%–58.29% of the total phenotypic variance in the different environments (Supplementary Table S1). Among these four traits, the QTL of BN exhibited the highest total average PVE of 47.57%, whereas those of BIN, BL, and BIL were 29.61%, 38.45%, and 43.50%, respectively (Supplementary Table S1). For the 53 BN-associated QTL, there was a major QTL region on Chr10:45,257,940..45,419,307 that displayed the highest PVE of 21.06% in 20JZ and 18.38% for the mean (Supplementary Figure S4; Supplementary Table S1). This region was identified as a major QTL associated with BIN (19 JZ, 20 JZ, and mean) and BL (20 JZ and mean) with a PVE >10% (Supplementary Figure S4; Supplementary Table S1). Regarding BIL, two major QTL regions with PVE > 10% were mapped to chromosome 13 (Chr13:38,262,556..38,389,319 and Chr13:41,863,394..41,887,491) and were separated by a distance of 3.47 Mb (Supplementary Table S1); however these two QTL regions were only detected from 20JZ and the mean, respectively (Supplementary Table S1).

Eight, five, three, and six QTL were identified for BN, BIN, BL, and BIL, respectively, using 3VmrMLM (Supplementary Table S4). These QTL were distributed across seven distinct chromosomes. The PVE of these four traits in the different environments (19JZ, 19SY, 20JZ, and 21SQ) ranged from 3.78% to 22.46% (Supplementary Table S1). Similar to the RTM-GWAS, 3VmrMLM identified more QTL for BN and yielded the highest total average PVE of 19.67% compared to that of BIN, BL, and BIL (Supplementary Table S1). Among all QTL identified by 3VmrMLM, a major QTL region associated with chromosome 10 (Chr10:45,257,940..45,322,107) explained 22.46%, 13.32%, 10.90%, and 5.78% of phenotypic variance in BN, BIN, BL, and BIL, respectively (Supplementary Figure S5; Supplementary Table S1). This region exhibited the highest PVE for BN, BIN, and BL but not for BIL. The QTL that explained the highest PVE for BIL (10.49%) was Chr11:24,394,808..24,461,847 that was mapped only from the 19SY (Supplementary Table S1).

### 3.3 Co-localized QTL and pleiotropic QTL

**FIGURE 2:**
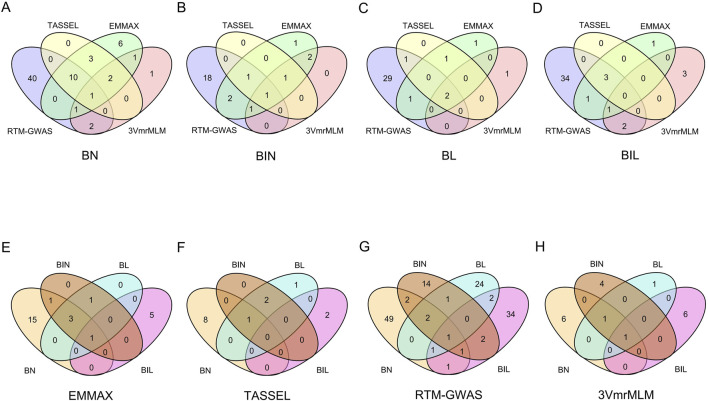
Co-localized QTL of BN, BIN, BL, and BIL in different environments (19JZ, 19SY, 20JZ, 21SQ) and mean. **(A–D)** QTL co-localized for the same trait in different methods **(E–H)**: Pleiotropy QTL for the same method.

LD between 251 QTL regions related to those four branch traits revealed that 166 QTL were co-localized (LD > 0.9), and these accounted for 66.14% of all QTL (Supplementary Table S3). Among these co-localized QTL regions, two major QTL regions (Chr10:43,366,437..47,047,085 and Chr11:10,609,377..11,279,201) were identified using four different methods and were associated with all four branch traits (Supplementary Table S3). Region Chr10:43,366,437..47,047,085 was co-localized with QTL identified using four different methods for three traits (BN, BIN, and BL) (Figures 2A–C; Supplementary Table S3). Furthermore, this region exhibited pleiotropic effects on the four branch traits according to two different methods (RTM-GWAS and 3VmrMLM) (Figures 2G, H). The other co-localization regions exerted a major effect. Chr11:10,609,377..11,279,201 was co-localized by four different methods in the BL, and this region was also pleiotropic for the four branch traits in EMMAX (Figures 2C, E). Additionally, there were another two QTL regions (Chr03:36,518,347..37,236,411 and Chr13:41,619,891..42,577,532) that revealed the co-localization of BN and BIL according to three different methods, thus indicating a pleiotropic effect (Figures 2A, D, G).

### 3.4 Candidate gene selection for the major QTL

**FIGURE 3:**
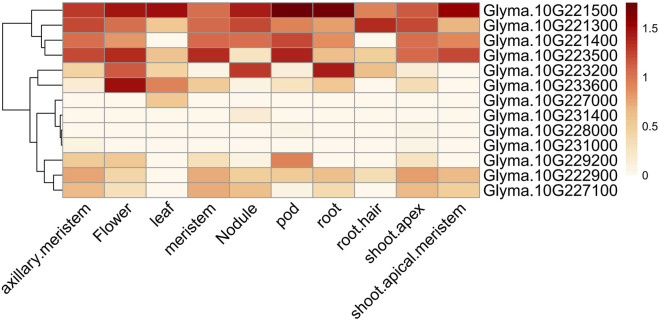
Spatial and temporal expression patterns of 13 candidate genes. The heat map illustrating the spatial and temporal expression profiles of 13 candidate genes, as derived from publicly available RNA-seq data. The data was normalized by log_10_(FPKM+1), where FPKM stands for Fragments Per Kilobase of exon model per Million mapped fragments.

The major QTL (Chr10:42,222,333..47,047, 085) with the highest PVE associated with the four branch traits was narrowed down to the interval Chr10:45,050,047..46,781,943,207 genes were identified, and 16 of them were observed to contain SNP variations resulting in stopgain and non-synonyms. Among these, 10 genes exhibited a genetic differentiation index (*F*_*ST*_) of greater than 0.6, thus indicating that they may have been subjected to domestication (Supplementary Table S6; Supplementary Table S7; Supplementary Figure S6). A spatial and temporal expression pattern analysis of the 10 domesticated genes revealed that nine genes were expressed either constitutively or specifically in certain tissues, and only one gene was not expressed (Figure 3). Two of the nine genes were annotated to encode unknown proteins (*Glyma.10G229200* and *Glyma.10G233600*), and one encoded a protein relevant to disease resistance (*Glyma.10G228000*) (Supplementary Table S8). Additionally, a region overlapped by all QTL mapped to this locus (Chr10:45,257,940..45,322,107) consisted of three genes that included *Glyma.10G221300*, *Glyma.10G221400*, and *Glyma.10G221500*. These nine genes are potential candidates for branch traits (Table 3).

**TABLE 3:** Gene function annotation of 9 candidate genes associated with four branch traits.

Gene	Annotation	Functional pathway	Homologue
*Glyma.10G221300*	S-adenosylmethionine carrier 1	Synthesis of ethylene and polyamines	AT4G39460.1
*Glyma.10G221400*	carboxypeptidase D, putative	catabolize various small acidic peptides and release small signaling molecules	AT1G71696.2
*Glyma.10G221500*	gigantea protein (GI)	growth and development of plants	AT1G22770.1
*Glyma.10G222900*	DHHC-type zinc finger family protein	Enhanced shoot branching	AT4G24630.1
*Glyma.10G223200*	Integrase-type DNA-binding superfamily protein	Ethylene transcription factor	AT5G61890.1
*Glyma.10G223500*	Cellulose synthase 6	Wall cellulose	AT5G64740.1
Glyma.10G227100	RING/FYVE/PHD zinc finger superfamily protein	plant growth, development, and responses to abiotic stresses	AT1G43770.2
*Glyma.10G231000*	Pectin lyase-like superfamily protein	Cell wall biosynthesis	AT3G48950.1
*Glyma.10G231400*	PPPDE putative thiol peptidase family protein	Ubiquitin signaling pathway	AT4G39460.1

### 3.5 Prediction accuracy of GS

**FIGURE 4:**
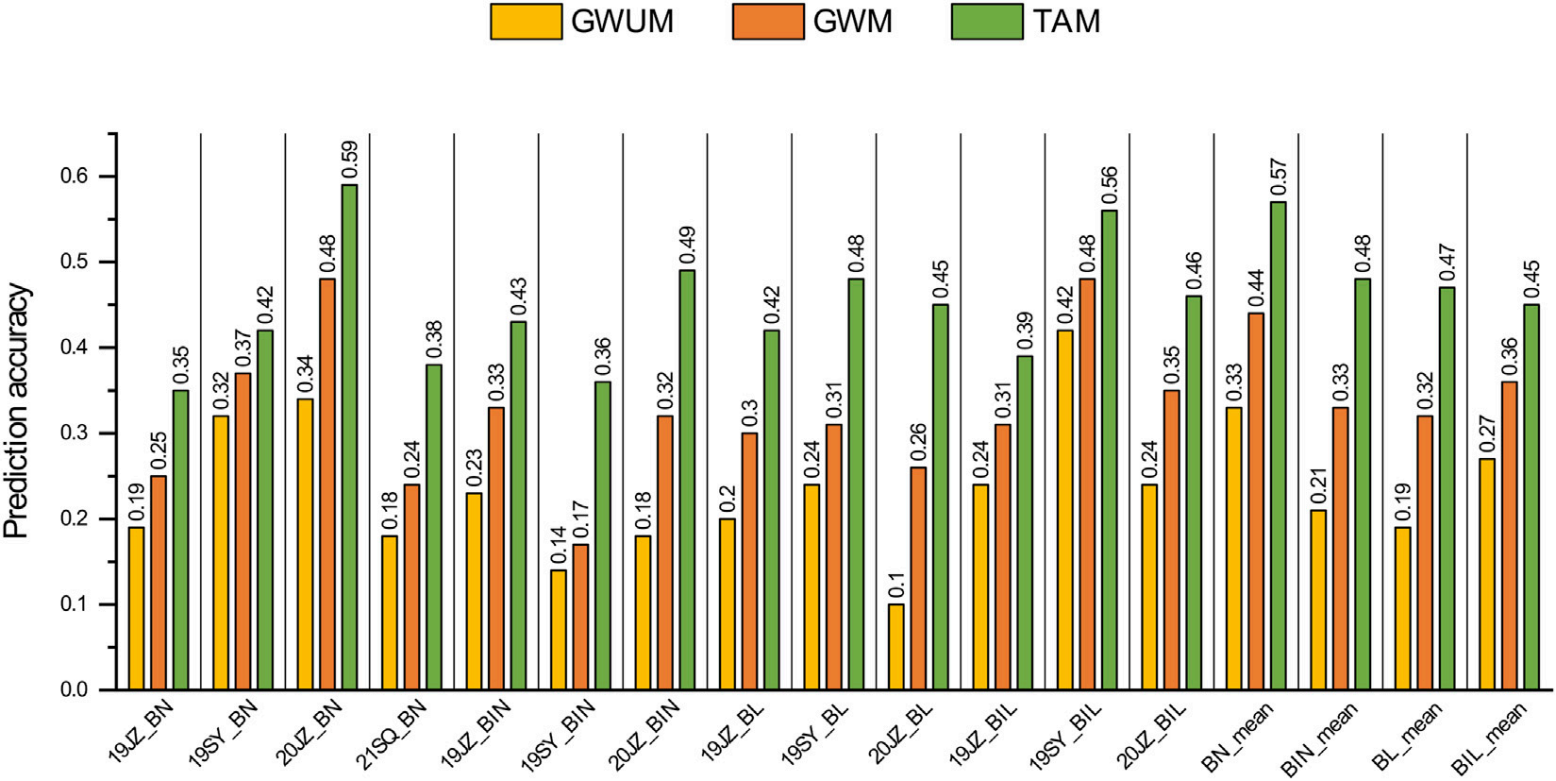
Prediction accuracy of genomic selection on BN, BIN, BL, and BIL. GWUM: 5698 Genome-wide uncorrelated markers, GWM: 6098 Genome-wide markers, TAM: 400 Trait associated markers.

The prediction accuracy for BN, BIN, BL and BIL was calculated using GBLUP, and prediction accuracies of 0.18–0.59, 0.14-0.49, 0.10-0.48, and 0.24-0.56 were identified, respectively, in the different environments (19JZ, 19SY, 20JZ, and 21SQ) of the three datasets. The values were 0.33–0.57, 0.21-0.48, 0.19-0.47, and 0.27-0.45, respectively, when the mean value of those traits was used as the phenotype (Figure 4). Comparatively, the prediction accuracy of the three traits with the exception of BIL was lower than the mean value in different environments. In terms of different marker sets, the prediction accuracy of TAM (Total associated markers) was the highest in either the mean or different environments with an average prediction accuracy of 0.46. The prediction accuracy of GWUM (Genome wide marker without associated markers) was the lowest, with an average prediction accuracy of 0.24. Compared to that of the TAM and GWM, the prediction accuracy of the TAM was improved by 15.28%–109.35%.

## 4 Discussion

### 4.1 QTL reliability was improved by using multiple QTL mapping methods

In different QTL mapping procedures, the number and effects of QTL are closely related to the population and model of QTL mapping (Fu et al., 2017). Localizing QTL using multiple QTL mapping methods can effectively reduce false-positive QTL and increase the confidence in major QTL (Sonah et al., 2015). In this study, 39, 17, 164, and 31 QTL related to those four branch traits were identified using EMMAX, TASSEL, RTM-GWAS, and 3VmrMLM, respectively, and 34, 17, 58 and 27 QTL identified by these four methods were co-localized with QTL identified by the other three methods, indicating a high repeatability of those methods. It worth noticing that all QTL detected by TASSEL were repeated in different environments, and although RTM-GWAS identified the most QTL, and very limited (35.4%) were repeated across environments, suggested TASSEL performed the best in our study and RTM-GWAS probably detected most of the missing heritability in different environments. The QTL identified by RTM-GWAS is much higher than those identified by other methods. This may be due to RTM-GWAS used multiple allelic variant markers and multi-locus models to comprehensively analyze the genetic composition of QTL in the population, which has been reported in previous studies (Gai and He, 2020). In addition, a total of 83 QTL overlapped in 25 QTL regions among all the QTL located by these methods, which may indicate that these regions are pleiotropic or one of the significant loci influencing the branch traits.

The 251 QTL mapped by those four different methods constitutes 143 unique regions of different branch traits (Supplementary Table S5). Among these unique QTL, Chr10:42,222,333..47,047,085 was the major QTL region that not only existed in all environments but also predominantly exhibited the highest PVE (Supplementary Table S1; Supplementary Table S5). Additionally, certain branching studies have localized this QTL in previous studies, thus indicating its high reliability (Ding, 2011; Li et al., 2008). Another major QTL region (Chr11:10,609,377..11,279,201) was co-localized by four different methods and was associated with four branch traits, thus suggesting that it is a reliable QTL (Supplementary Table S1; Supplementary Table S3; Supplementary Table S5).

### 4.2 Candidate gene for branch traits

Among the 207 genes in the region of Chr10:45,050,047..46,781,943 that were analyzed by SNP variant analysis, *F*_*ST*_, and spatial temporal specific expression analysis, nine genes may be candidate genes for soybean branch number, including seven genes encoding known proteins and two genes encoding proteins of unknown function. Of the seven genes encoding known proteins, six may be associated with soybean branch traits, with the exception of a disease-associated gene. *Glyma.10G222900* encodes a DHHC-type zinc finger family protein that may control plant growth and development, including that of branches (Xiang et al., 2010). The integrase-type DNA-binding superfamily protein encoded by *Glyma.10G223200* may exhibit high homology to AtERF114 and regulate plant branches and structures in an auxin-dependent manner (Lyu et al., 2022). *Glyma.10G223500* regulates cellulose content by encoding cellulose synthase 6 (Endler et al., 2016). *Glyma.10G227100* encodes the RING/FYVE/PHD zinc-finger superfamily protein that is associated with plant growth, development, and abiotic stress responses (Han et al., 2022). The pectin lyase-like superfamily protein is encoded by *Glyma.10G231000* and plays a key role in cell wall biosynthesis and organization (Han et al., 2018; Yu et al., 2021). The PPPDE putative thiol peptidase family protein is encoded by *Glyma.10G231400* and regulates the ubiquitin signaling pathway (Liu et al., 2020). Additionally, three genes in the region Chr10:45,257,940..45,322,107 were also correlated with branch traits with a high probability. The Family S-adenosylmethionine carrier 1 encoded by *Glyma.10G221300* (S-adenosylmethionine) is associated with the synthesis of ethylene and polyamines (Yang et al., 2023). *Glyma.10G221400* encodes a putative protein known as carboxypeptidase D. The *PLASTOCHRON3 (PLA3)/GOLIATH (GO)* gene encoding this protein in rice is capable of converting rachis branches into shoots (Kawakatsu et al., 2009). The Gigantea protein encoded by *Glyma.10G221500* regulates the growth and development of plants and is involved in the regulation of flowering time in soybeans (Wong et al., 2009; Patnaik et al., 2023; Watanabe et al., 2011). Interestingly, some genes related to other traits, e.g., flowering time, maturation time, were reported to be colocalized within those regions (Xia et al., 2021). It is reported that the tillering in rice was influenced by light signal via strigolactone pathway (Xie et al., 2020), suggested a possible role of gene involved in the light receiving participated in branching in soybean.

### 4.3 Genomic prediction

Genomic selection is a powerful tool for plant and animal improvement, however, it has not yet been applied to soybean branch traits. The identification of 99, 43, 50, and 59 QTL associated with BN, BIN, BL, and BIL, respectively, suggests that these four traits are quantitative traits controlled by multiple loci. These four branch traits exhibited high heritability (>0.75), thus suggesting the possibility of higher prediction accuracy for GS (Lan et al., 2020). Based on the three genomic marker sets, GS studies examining BN, BIN, BL, and BIL revealed that the prediction accuracy of GWUM was lower than that of GWM, whereas the prediction accuracy of TAM was higher than that of GWM, thus indicating that marker density does not always improve prediction accuracy (Xavier et al., 2016). Retaining 400 SNPs relevant to the four branch traits was an effective approach to improve the prediction accuracy of different traits, possibly due to the observation that the reduction in SNPs decreased the background noise with no or low effect. This result is consistent with those of previous studies, thus indicating that most of the QTL identified in this study were effective (Xiong et al., 2023).

In this study, a batch of different QTL related to branch traits was identified using different methods, which assisted the marker development and marker assisted selection. At the meanwhile, genomic selection model was constructed based on the QTL mapped as well, which could be applied in the branch traits selection in the breeding populations and accelerate the breeding process. And candidate genes identified in this study provided solid foundation towards the gene cloning and molecular mechanism clarification of branch related traits.

## 5 Conclusion

A total of 99, 43, 50, and 59 QTL were identified for BN, BIN, BL and BIL respectively, based on which genomic selection model was constructed with prediction accuracy of larger than 0.48. For the major QTL with highest PVE, nine candidate genes were screened out for further study. This study provided a deep view of genetic mechanism underlying branch related traits, provided a beneficial trial of genomic selection on soybean branch related traits, which could be applied in breeding programs.

## Data Availability

The data presented in the study are deposited in the Soybean Functional Genomics and Breeding repository, direct access to data through link https://sfgb.rmbreeding.cn/about/data.

## Abbreviations

BIL, Branch Internode Length; BIN, Branch Internode Number; BL, Branch Length; BN, Branch Number; CTAB, Cetyltrimethylammonium Bromide; EMMAX, Efficient Mixed-Model Association Expedited; FST, Fixation Index; GBLUP, Genomic Best Linear Unbiased Predictor; GS, Genomic Selection; GWM, Genome-Wide Markers; GWUM, Genome-Wide Unassociated Markers; IQR, Interquartile Range; LD, Linkage Disequilibrium; MLM, Mixed Linear Model; PVE, Phenotypic Variance Explained; QTL, Quantitative Trait Loci; RIL, Recombinant Inbred Line; RTM-GWAS, Restricted Two-Stage Multi-Locus Genome-Wide Association; SNP, Single Nucleotide Polymorphism; SNPLDBs, SNP Linkage Disequilibrium Blocks; TAM, Trait-Associated Markers; TASSEL, Trait Analysis by Association, Evolution and Linkage; ZD 41, Zhongdou 41; 3VmrMLM, Three-Variance Component Multi-Locus Random SNP Effect Mixed Linear Model.
