# Supplementary material for: Identification of QTL for branch traits in soybean (Glycine max L.) and its application in genomic selection
Source: Front Genet. 2025 Mar 3;16:1484146. doi: 10.3389/fgene.2025.1484146 (PMC11911462; doi:10.3389/fgene.2025.1484146)
Supplement: Supplementary file 1 [file DataSheet1.PDF]

Supplementary Figure S1

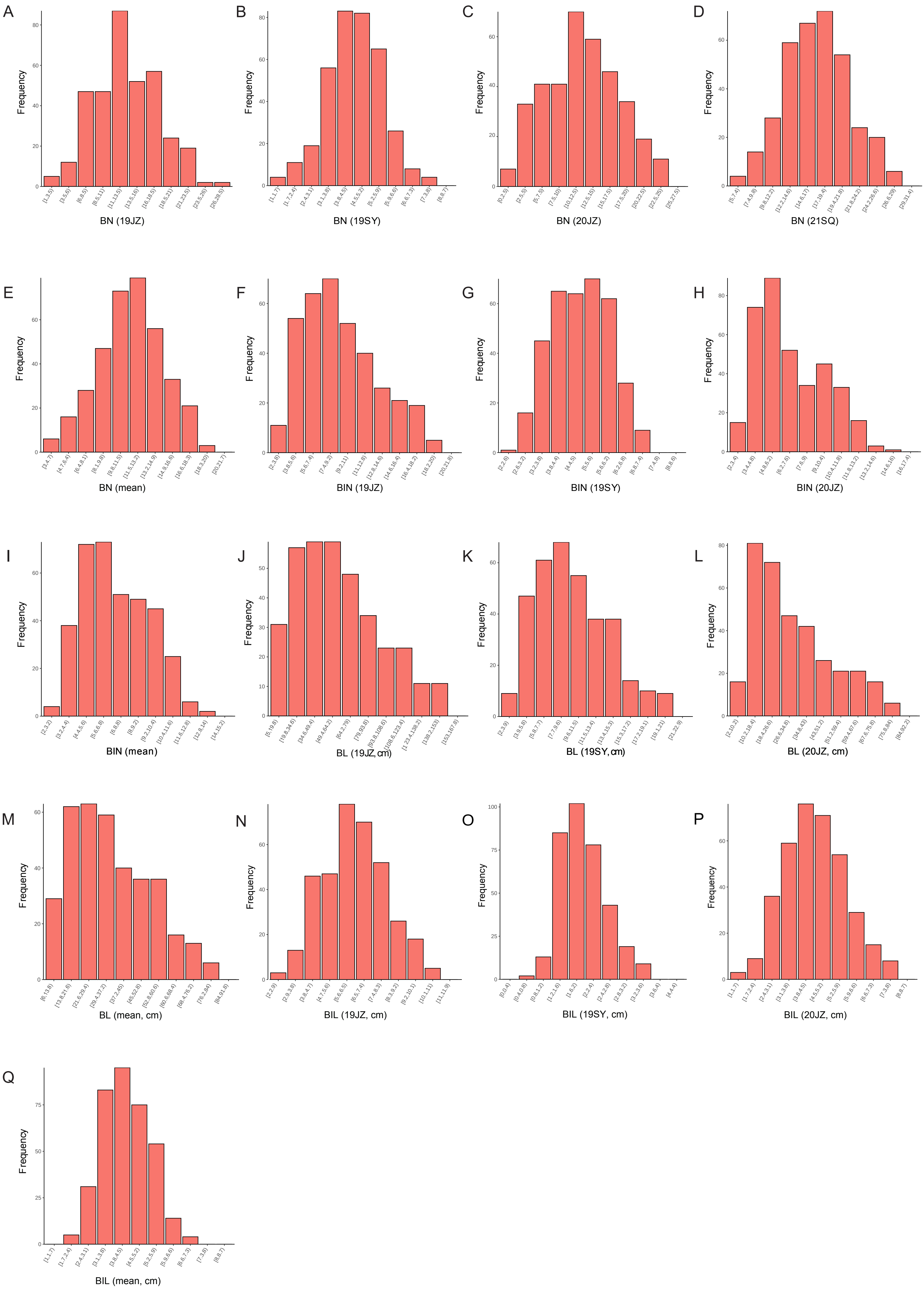

Supplementary Figure S1. Normal distribution of 4 traits under 4 environments with mean.

Supplementary Figure S2

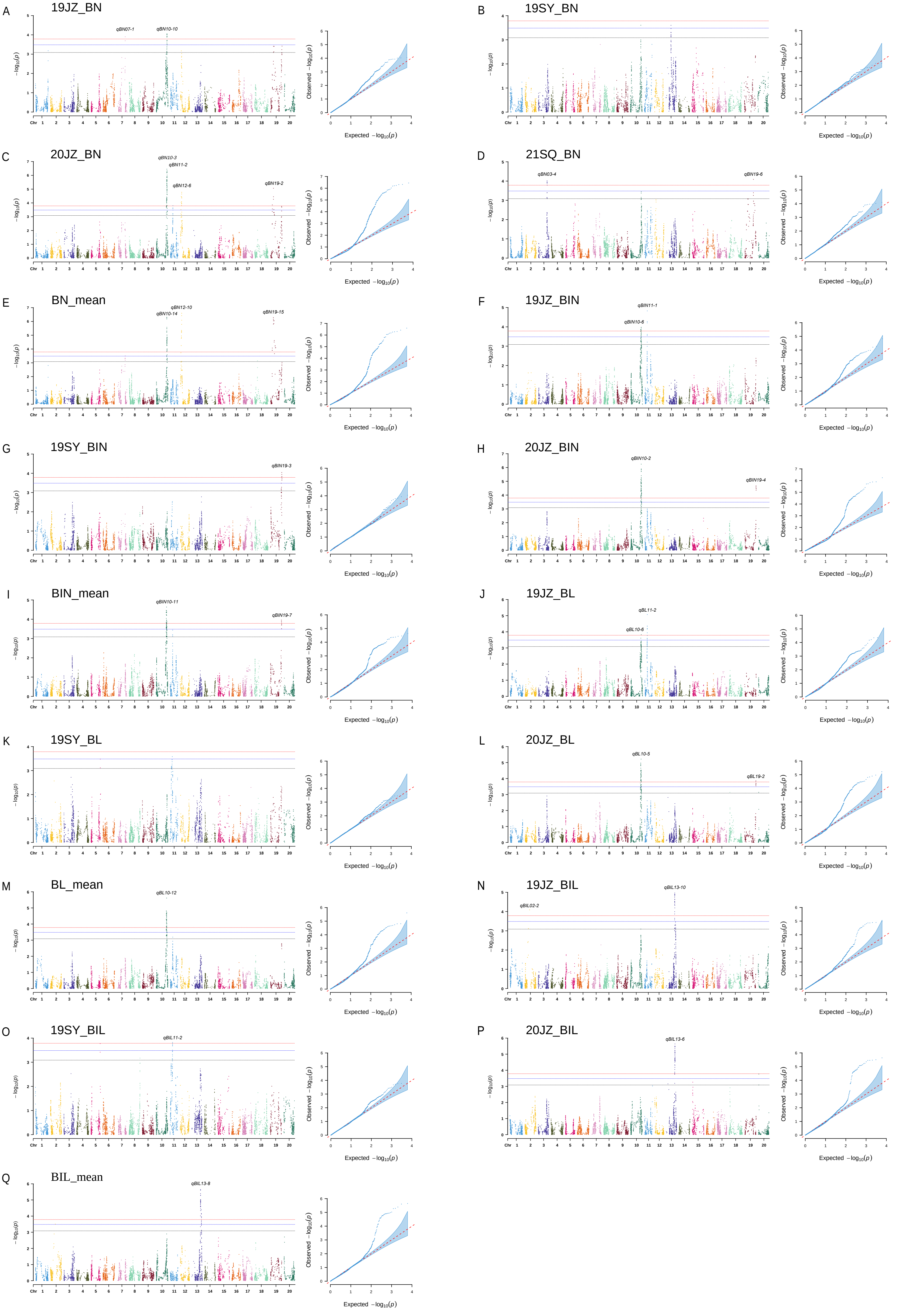

Supplementary Figure S2. Manhattan and QQ plot of EMMAX.The most significance level of Manhattanwas set to -lg(1/m). where m is the marker number.

Supplementary Figure S3

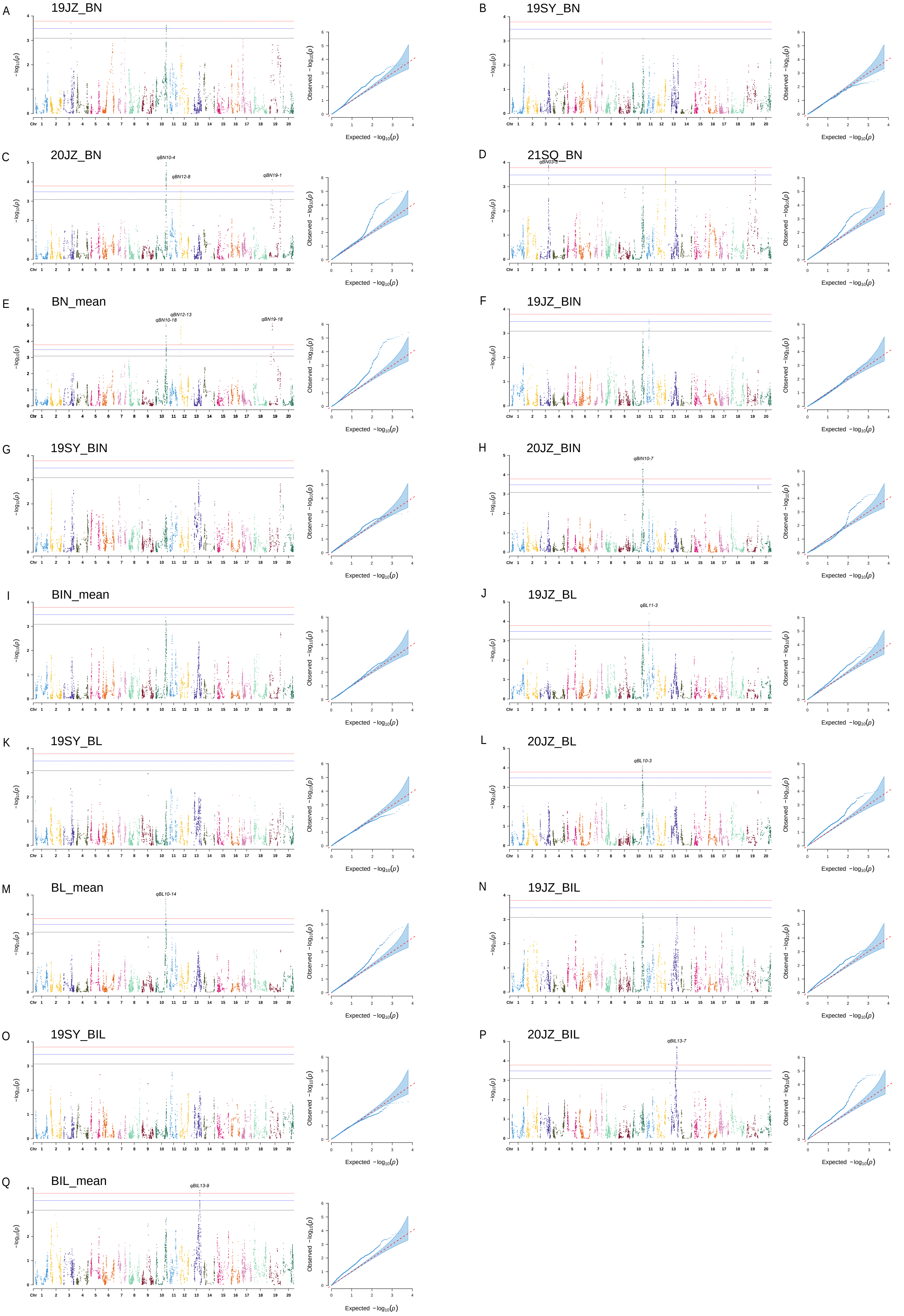

Supplementary Figure S3. Manhattan and QQ plot of TASSEL.

Supplementary Figure S4

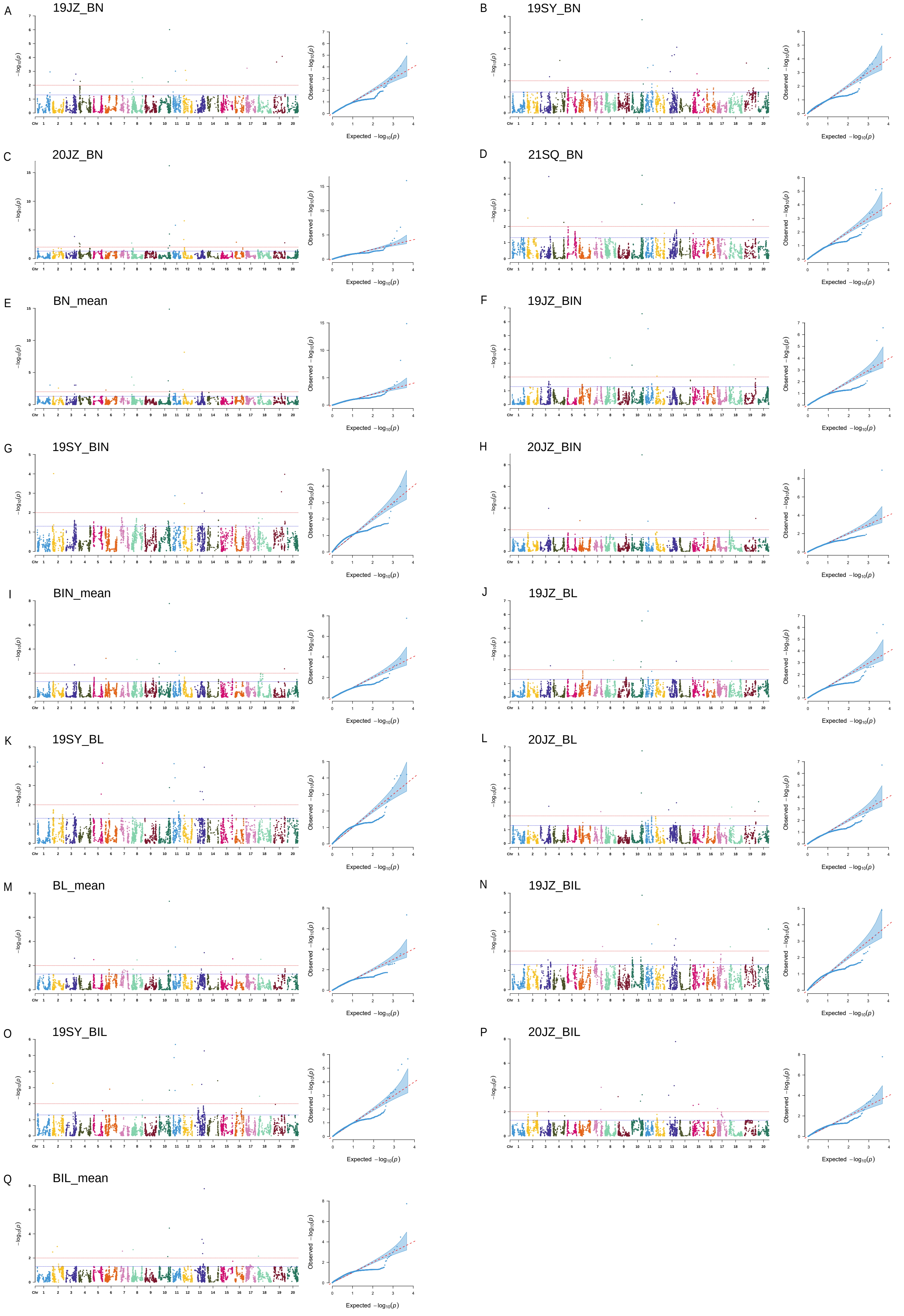

Supplementary Figure S4. Manhattan and QQ plot of RTM-GWAS.

Supplementary Figure S5

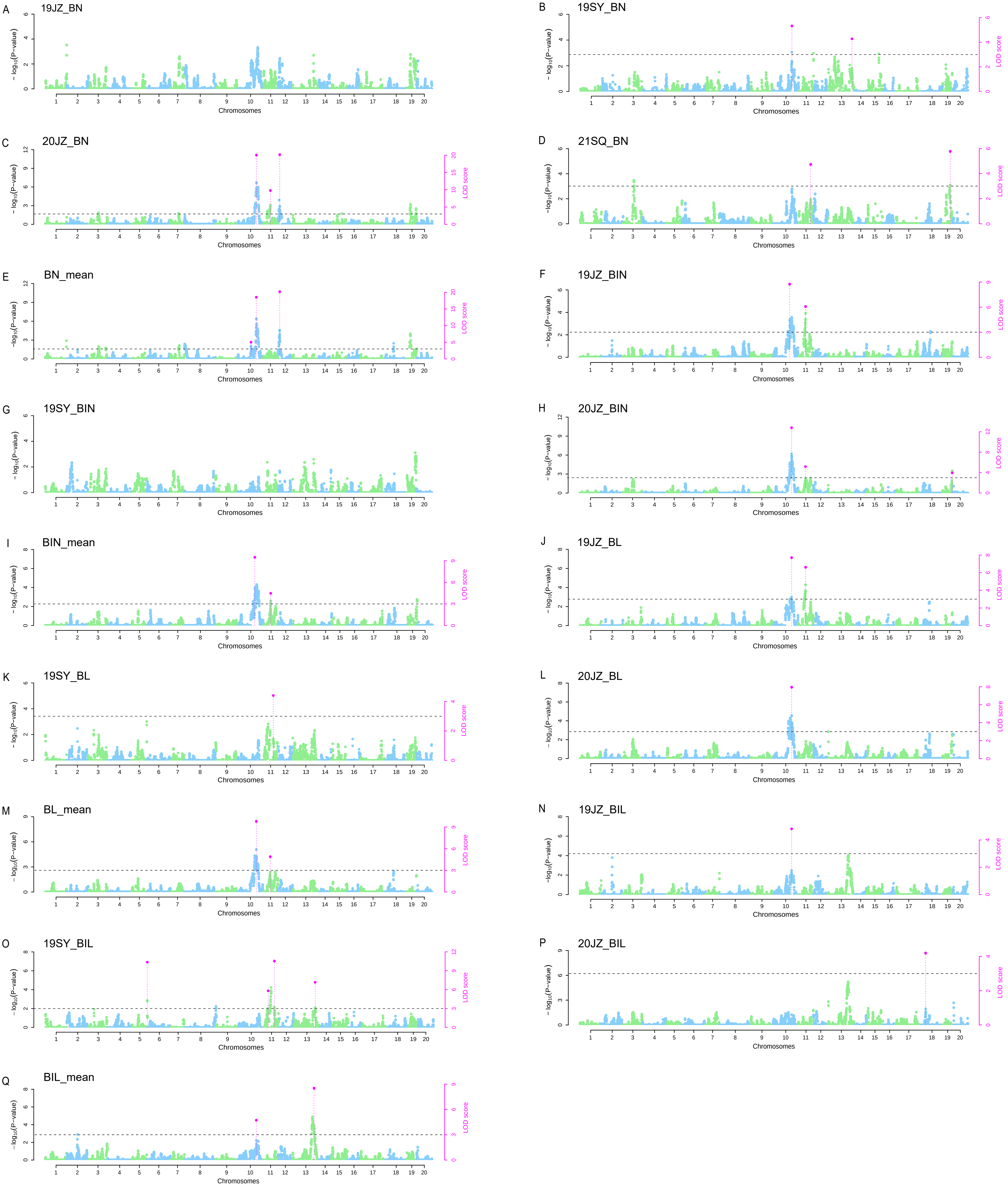

Supplementary Figure S5. Manhattan plot of 3VmrMLM.

Supplementary Figure S6

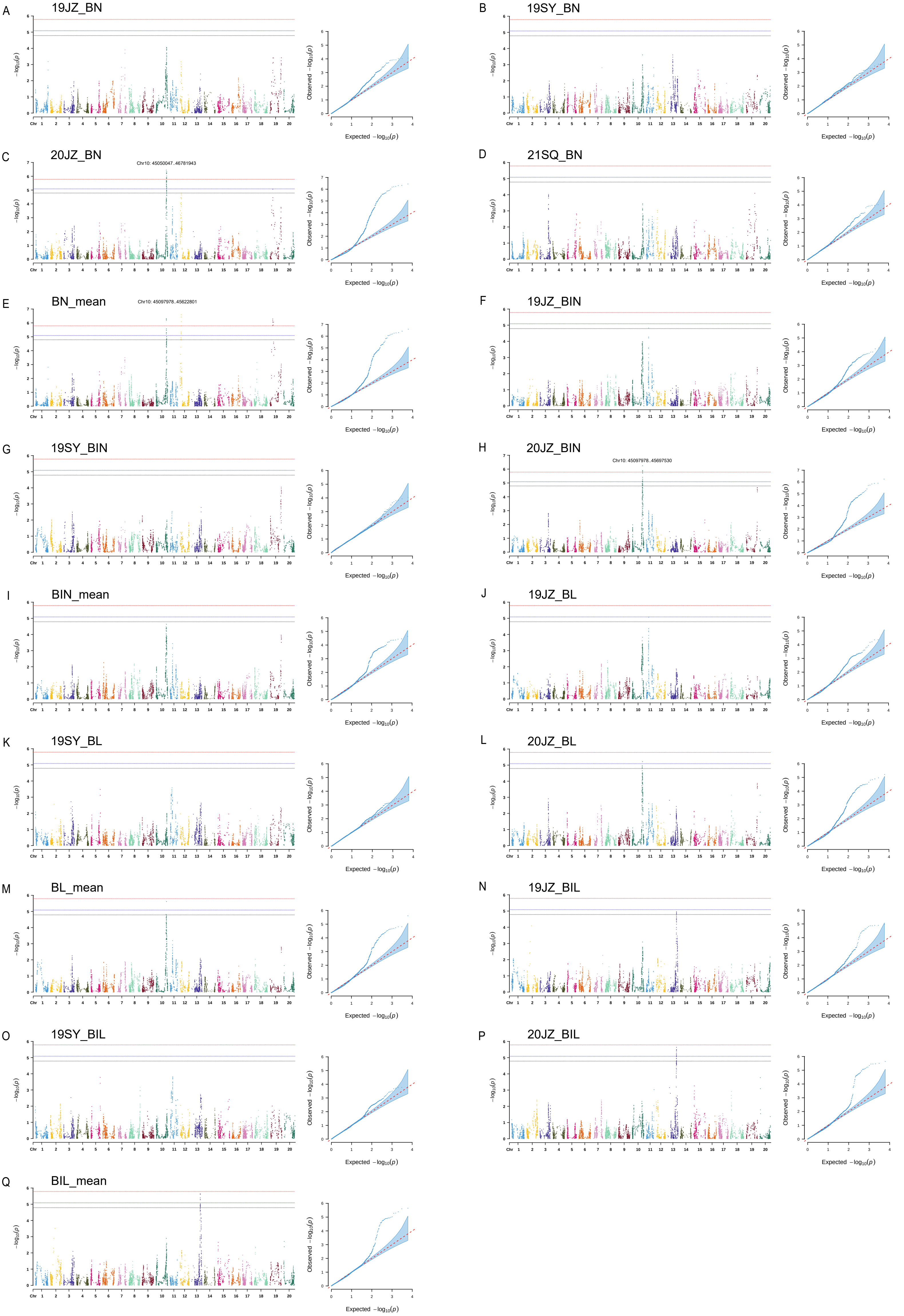

Supplementary Figure S6. Manhattan and QQ plot of EMMAX. The most significance level of Manhattan was set to -lg(0.01/m), where m is the marker number.
